# Development and External Validation of the International Early Warning Score for Improved Age- and Sex-Adjusted In-Hospital Mortality Prediction in the Emergency Department

**DOI:** 10.1097/CCM.0000000000005842

**Published:** 2023-03-23

**Authors:** Bart Gerard Jan Candel, Søren Kabell Nissen, Christian H. Nickel, Wouter Raven, Wendy Thijssen, Menno I. Gaakeer, Annmarie Touborg Lassen, Mikkel Brabrand, Ewout W. Steyerberg, Evert de Jonge, Bas de Groot

**Affiliations:** 1 Department of Emergency Medicine, Leiden University Medical Center, Leiden, The Netherlands.; 2 Department of Emergency Medicine, Máxima Medical Center, Veldhoven, The Netherlands.; 3 Institute of Regional Health Research, Center South-West Jutland, University of Southern Denmark, Esbjerg, Denmark.; 4 Department of Emergency Medicine, Odense University Hospital, Odense, Denmark.; 5 Department of Emergency Medicine, University Hospital Basel, University of Basel, Basel, Switzerland.; 6 Department of Emergency Medicine, Catharina Hospital Eindhoven, Eindhoven, The Netherlands.; 7 Department of Emergency Medicine, Admiraal de Ruyter Hospital, Goes, The Netherlands.; 8 Department of Emergency Medicine, Hospital of South-West Jutland, Esbjerg, Denmark.; 9 Department of Public Health, Erasmus University Medical Center, Rotterdam, The Netherlands.; 10 Department of Biomedical Data Sciences, Leiden University Medical Center, Leiden, The Netherlands.; 11 Department of Intensive Care Medicine, Leiden University Medical Center, Leiden, The Netherlands.; 12 Department of Emergency Medicine, Radboud University Medical Center, Nijmegen, The Netherlands.

**Keywords:** early warning score, emergency medicine, geriatrics, physiologic monitoring, sex differences

## Abstract

**Objectives:**

Early Warning Scores (EWSs) have a great potential to assist clinical decision-making in the emergency department (ED). However, many EWS contain methodological weaknesses in development and validation and have poor predictive performance in older patients. The aim of this study was to develop and externally validate an International Early Warning Score (IEWS) based on a recalibrated National Early warning Score (NEWS) model including age and sex and evaluate its performance independently at arrival to the ED in three age categories (18–65, 66–80, > 80 yr).

**Design:**

International multicenter cohort study.

**Setting:**

Data was used from three Dutch EDs. External validation was performed in two EDs in Denmark.

**Patients:**

All consecutive ED patients greater than or equal to 18 years in the Netherlands Emergency department Evaluation Database (NEED) with at least two registered vital signs were included, resulting in 95,553 patients. For external validation, 14,809 patients were included from a Danish Multicenter Cohort (DMC).

**Measurements and Main Results:**

Model performance to predict in-hospital mortality was evaluated by discrimination, calibration curves and summary statistics, reclassification, and clinical usefulness by decision curve analysis. In-hospital mortality rate was 2.4% (*n* = 2,314) in the NEED and 2.5% (*n* = 365) in the DMC. Overall, the IEWS performed significantly better than NEWS with an area under the receiving operating characteristic of 0.89 (95% CIs, 0.89–0.90) versus 0.82 (0.82–0.83) in the NEED and 0.87 (0.85–0.88) versus 0.82 (0.80–0.84) at external validation. Calibration for NEWS predictions underestimated risk in older patients and overestimated risk in the youngest, while calibration improved for IEWS with a substantial reclassification of patients from low to high risk and a standardized net benefit of 5–15% in the relevant risk range for all age categories.

**Conclusions:**

The IEWS substantially improves in-hospital mortality prediction for all ED patients greater than or equal to18 years.

**KEY POINTS**

**Question:** Developing and externally validate an age and sex adjusted early warning score (the International Early Warning Score [IEWS]) to improve prediction of the in-hospital mortality risk at arrival to the emergency department (ED).

**Findings:** This multicenter cohort study shows that the IEWS performs significantly better compared with the widely adopted National Early warning Score (NEWS) for the prediction of in-hospital mortality in ED patients of all age categories in a development and external validation cohort. Compared with using NEWS, the IEWS would identify 5–15% additional true deaths without increasing the number of false positive predictions. Additionally, young patients are more often correctly recognized as low risk for in-hospital mortality, while older patients are more often correctly recognized as high risk.

**Meaning:** The IEWS can more accurately predict in-hospital mortality than NEWS at arrival to the ED for all adult patients. Therefore, it may improve decision support in the ED, especially for older patients, who are often at higher risk.

Early Warning Scores (EWSs) are widely used prediction tools to early detect clinical deterioration of patients and trigger intensive care consultation (1–4). By aggregating points for the degree of abnormality of each vital sign, EWSs provide a likelihood for mortality, which should trigger the nurse or physician to get help or to intensify treatment. These scores are widely used in many settings and they are mandatory as a standard of care in the United Kingdom (5). The National Early warning Score (NEWS) in particular has been widely implemented and is the most frequently used score to help identify critically ill patients early (4–7).

Some limitations of the NEWS and other EWSs exist. Calibration of NEWS predictions is poor with relative overestimation of risk in younger emergency department (ED) patients and underestimation of risk in older ED patients (8–10). NEWS assigns 0 to 3 points for all vital signs implying that all vital signs have similar predictive value, which has been shown to be unfounded (11, 12). Furthermore, important risk differences exist between men and women at arrival to the ED (13, 14). Nonetheless, most studies do not test the performance of EWSs at older age or include sex differences (1, 15). As a result, using NEWS may cause serious disadvantages for patient care and wrong treatment or disposition decisions.

The aim of this study was to develop and externally validate an International Early Warning Score (IEWS), by recalibrating NEWS including age and sex, to improve in-hospital mortality prediction at arrival to the ED.

## METHODS

### Study Design and Setting

This international multicenter cohort study is based on existing cohorts and reporting adheres to the Transparent Reporting of a multivariable model for Individual Prognosis or Diagnosis (TRIPOD) guidelines for prognostic modeling studies (16). The Netherlands Emergency department Evaluation Database (NEED) was used as development cohort, consisting of three hospitals in the Netherlands. The NEED is the national quality registry for EDs in the Netherlands and contributes to the improvement of transparency and quality of ED care in the Netherlands by supplying reliable data to the participating centers (see www.stichting-need.nl) (11). Data were prospectively collected and reviewed retrospectively. Data from the three sites spanned slightly different periods: data from one tertiary center (Leiden University Medical Center) included visits between January 1, 2017, and June 8, 2019, and data from the two level II emergency centers (Medical Center Leeuwarden and Catharina Hospital Eindhoven) were from January 1, 2019, to January 12, 2020, and from January 1, 2017, to December 31, 2019, respectively.

For external validation, we used the Danish Multicenter Cohort (DMC) which has been described previously (10, 17). These data were not only from a different setting but also from a different period to strengthen our validation. Patients were consecutively sampled in relation to previous prospective studies at two level II emergency centers: University Hospital of Southwest Jutland: (October 2, 2008, to February 12, 2009; February 23, 2010, to May 26, 2010; June 1, 2012, to November 1, 2011; April 24, 2013, to December 9, 2013) and Lillebaelt Hospital (January 1, 2010, to June 30, 2010).

### Ethical Considerations

In the Netherlands, the study was approved by the medical ethics committee of the Máxima MC on February 2, 2021 (ref number: Institutional Review Board N21.007). Under Danish law, retrospective registry studies are exempt from the need for approval by an ethics committee (18). The study has been performed in accordance with the Helsinki declaration of 1975.

### Selection of Participants

All consecutive ED patients of greater than or equal to 18 years were included in this study. Patients were excluded in the NEED if none or only one vital sign (systolic blood pressure [SBP], heart rate, peripheral oxygen saturation, respiratory rate, or temperature) were registered, as vital signs were considered missing not at random which prevented the possibility for imputation (**Table E1**, http://links.lww.com/CCM/H313). Both studies collected data prospectively, but the DMC did so based on a prospective study design and the NEED was based on a registry. Hence, the missing data mechanisms differed for DMC (**Table E2**, http://links.lww.com/CCM/H313). Here, patients were excluded if neither SBP nor pulse were recorded as these observations were missing not at random, that is, unrelated to any of the observed variables, including outcomes.

### Data Collection

Demographic data were extracted from registers for both the NEED and DMC. Implausible physiologic values were considered missing. Vital signs were recorded by a nurse in triage before ED treatment as described previously for the NEED (11), and for the DMC (10, 17). The first initial set of vital signs was registered before treatment.

NEWS aggregates seven vital signs (**Table 1**) (4). The NEWS was calculated for each patient (0–23 points) (4). The collected Glasgow Coma Scale was converted to an Alert, Verbal, Pain, Unresponsive (AVPU) score (10).

**TABLE 1.** The National Early Warning Score

Points	3	2	1	0	1	2	3
Respiratory rate (breaths/min)	≤ 8		9–11	12–20		21–24	≥ 25
Peripheral oxygen saturation (%)	≤ 91	92–93	94–95	≥ 96			
Supplemental oxygen		Yes		No			
Temperature (°C)	≤ 35.0		35.1–36.0	36.1–38.0	38.1–39	≥ 39.1	
Systolic blood pressure (mm Hg)	≤ 90	91–100	101–110	111–219			≥ 220
Pulse (beats/min)	≤ 40		41–50	51–90	91–110	111–130	≥ 131
Level of consciousness				Alert			Verbal, pain, or unresponsive

### Outcome

The primary outcome was in-hospital mortality (including death in the ED). This outcome measure allowed us to compare our findings with previous studies (10, 19). In the NEED, outcome information was registered and collected from the minimal dataset. In the DMC, information regarding mortality was collected retrospectively from the Danish Civil Registration System and the Danish National Patient Register.

### Sample Size Estimation

See **Appendix 1** (http://links.lww.com/CCM/H313).

### Data Analyses

#### Descriptive Analyses

Data were presented as mean (sd) if normally distributed and median (interquartile range [IQR]) if skewed.

#### Main Statistical Analyses

Predictive performance of NEWS and a recalibrated NEWS were evaluated in three age categories (18–65, 66–80, > 80 yr). These age categories were chosen based on previous age stratification (10, 11). Prior to analyses, we assessed nonlinearity of age in univariable logistic regression and explored nonlinear terms (quadratic and restricted cubic splines) for best fit. Because patients were included if at least two vital signs were registered, missing data in the NEED were substituted by multiple imputation to reduce information bias described in Appendix 1 (http://links.lww.com/CCM/H313) (20).

For each imputation set, we calculated the NEWS. We used the vital sign categories as used in the NEWS as ordinal variables to fit the new model to prevent introducing thresholds different to those professionals are used to in current clinical practice with NEWS. We fitted the model NEWS + age + sex on the imputed data by multivariable logistic regression and, in a backwards selection approach, tested one-way and two-way interactions among predictors and found none of sufficient impact to include in the revised model. After deciding on recalibration, points were assigned and rounded to a recalibrated NEWS score based on a nomogram presentation, that is, regression coefficients (21). Points were rounded to nearest integer.

Predictive performance was compared in all three age categories of NEWS, recalibrated NEWS + age, and the recalibrated NEWS + age + sex using area under the receiving operating characteristic (AUROC) with 95% CIs and calibration plots. We averaged regression coefficients and intercepts across imputed sets to incorporate variance introduced by the imputation procedure. The best of the two recalibrated models was named the IEWS.

To compare the net benefit of IEWS with NEWS, decision curves are presented (22). This plots net-benefit at a range of risk thresholds for in-hospital mortality with the trade-off of benefit (true positive proportion) and harms (false positive proportion) on the same scale, adjusted by an appropriate exchange rate (23). Because risk thresholds may differ by age group, separate decision curves were produced. To demonstrate how IEWS classifies patients differently than NEWS, a reclassification table was produced in which patients were allocated to low risk, medium risk, or high risk subsets, stratified by outcome. In this example, we decided that the threshold from low to medium risk was two times the baseline risk and medium to high risk was three times the baseline risk (mean in-hospital mortality for patients with a NEWS < 4 points) per age category.

#### Internal and External Validation

See Appendix 1 (http://links.lww.com/CCM/H313).

All analyses were performed in R statistical software packages dplyr (v1.0.7) (24), rms (v6.2 (25), and mice (v45) (26). A *p* value of less than 0.05 was considered as statistically significant.

## RESULTS

### Patient Characteristics

In total, 95,553 patients could be included for analyses from the NEED with mean age 60.1 years (sd, 19.4 yr) and 50.3% male patients. Patient characteristics are described in **Table E3** (http://links.lww.com/CCM/H313). Excluded patients had lower in-hospital mortality and fewer ICU admissions than the included patients (Table E1, http://links.lww.com/CCM/H313). For external validation, a total of 14,809 patients were included. They had a mean age of 63 years (sd, 20 yr), 51.9% were male. Patient characteristics in DMC were comparable to the NEED (**Table E4**, http://links.lww.com/CCM/H313).

### Main Results

Age was used as a linear spline with no age effect assumed below 40 years based on its fit and association with mortality (**Fig. E1**, http://links.lww.com/CCM/H313). A nomogram for the recalibrated NEWS plus age and sex was presented (**Fig. E2**, http://links.lww.com/CCM/H313).

**Figure 1.**
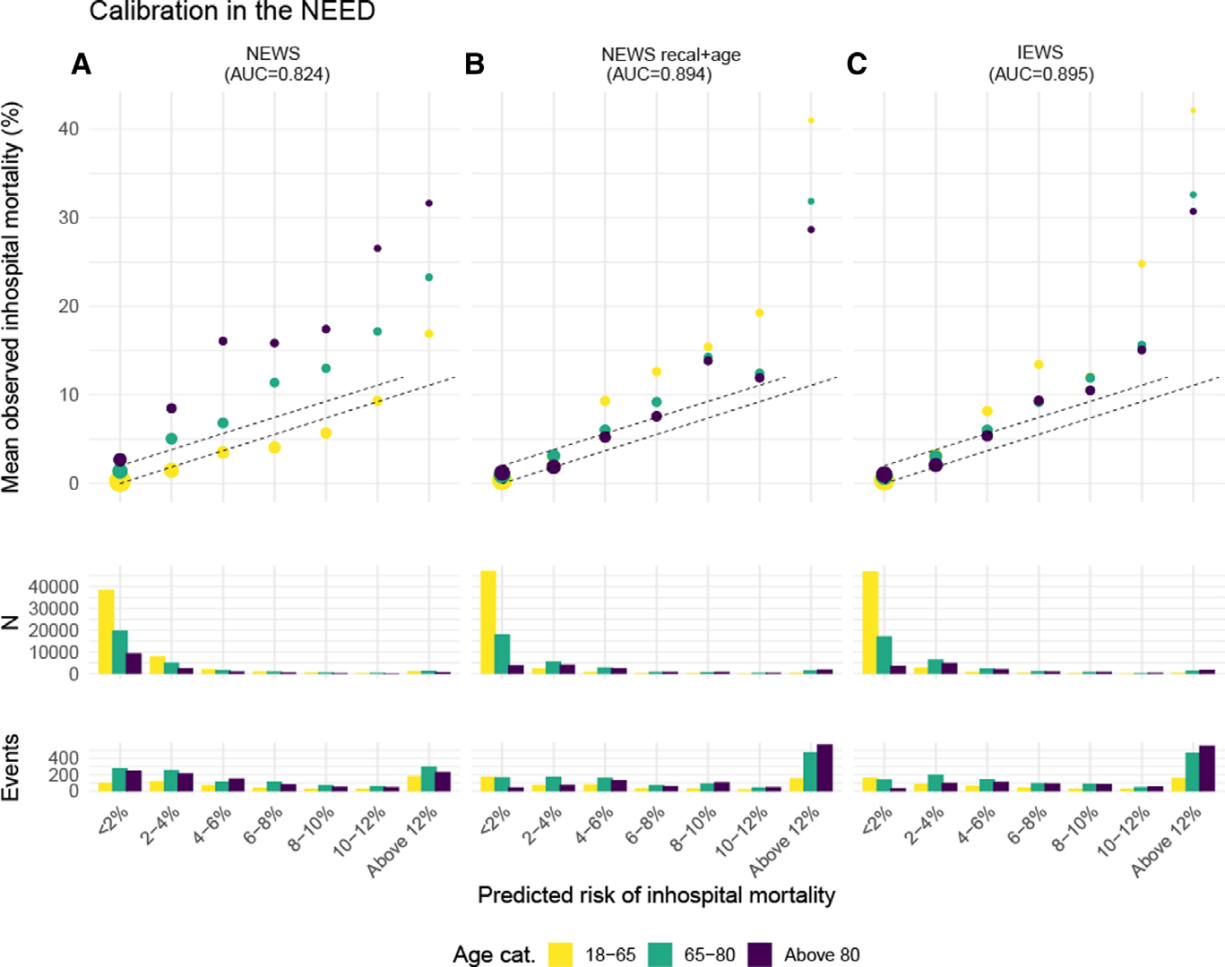
This figure shows the internal calibration plots for the three different models evaluated in the development cohort, the Netherlands Emergency department Evaluation Database (NEED), with the outcome in-hospital mortality. Calibration improves visually from the left plot (NEWS) to the right plot (IEWS). Also, the distribution of patients and events are described per predicted risk category. Internal calibration plots for the NEED for the NEWS (**A**), a recalibrated NEWS + age (**B**), and a recalibrated NEWS + age + sex (the IEWS) (**C**). The predicted in-hospital mortality was categorized in steps of 2% in the relevant risk range. Calibration was assessed in three different age categories (18–65, 66–80, > 80 yr). The *dotted lines* represent ideal calibration. The size of the *dots* indicates the precision of the estimate for observed in-hospital mortality in each risk group, the larger, the higher the precision based on the inverse of the sd. Below the calibration figures are the distribution of patients and outcomes presented for all three scores in histograms. AUC = area under the curve.

**Figure 2.**
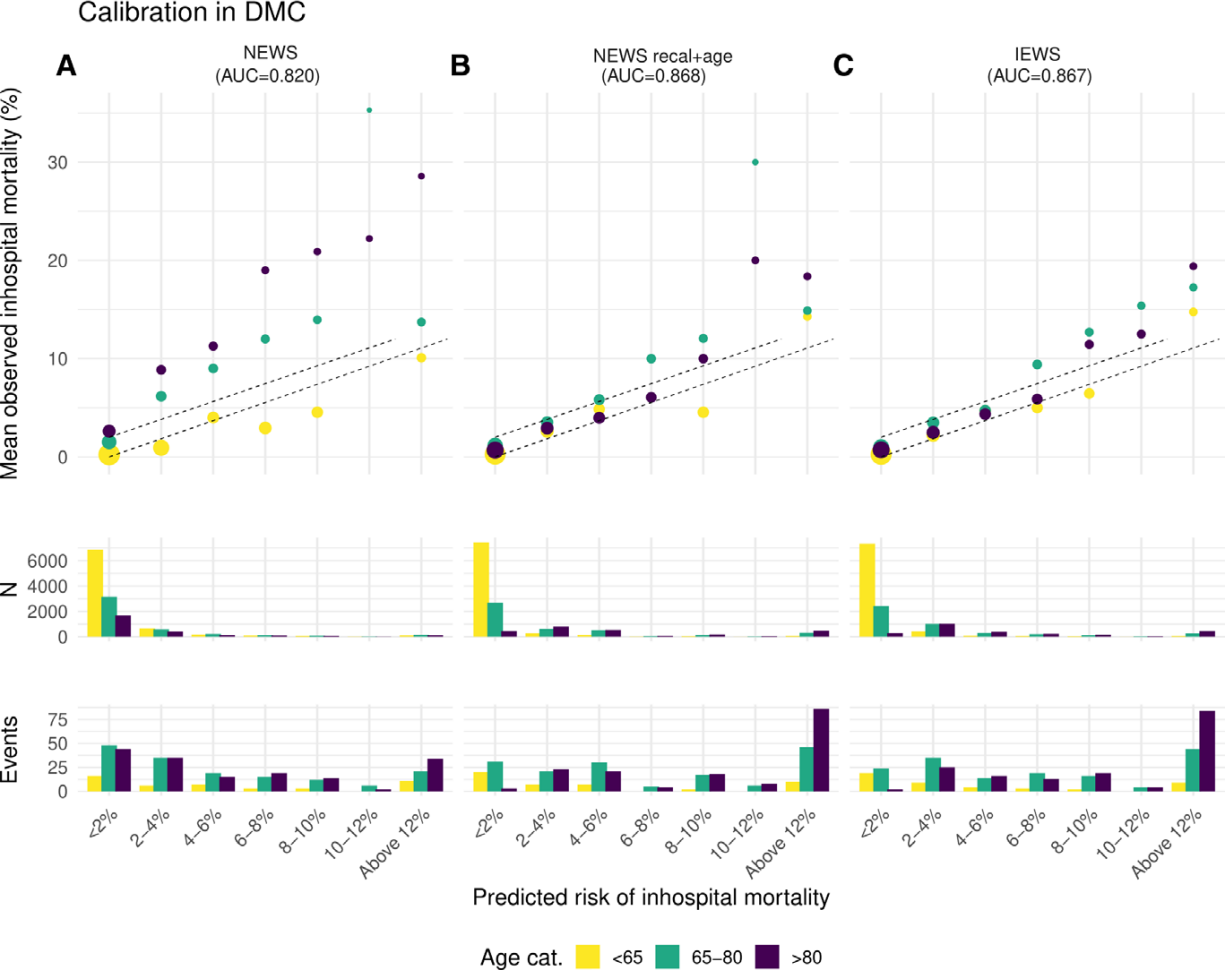
This figure shows the external calibration plots for the three different models evaluated in the validation cohort, the Danish Multicenter Cohort (DMC), with the outcome in-hospital mortality. Calibration improves from the left plot (NEWS) to the right plot (IEWS). Also, the distribution of patients and events are described per predicted risk category. External calibration plots for the DMC for the NEWS (**A**), a recalibrated NEWS + age (**B**), and a recalibrated NEWS + age + sex (the IEWS) (**C**). The predicted in-hospital mortality was categorized in steps of 2% in the relevant risk range. Calibration was assessed in three different age categories (18–65, 66–80, > 80 yr). The *dotted lines* represent ideal calibration. The size of the *dots* indicates the precision of the estimate for observed in-hospital mortality in each risk group, the larger, the higher the precision based on the inverse of the sd. Below the calibration figures are the distribution of patients and outcomes presented for all three scores in histograms. AUC = area under the curve.

Based on the nomogram, points were assigned for a recalibrated NEWS + age score and a recalibrated NEWS + age + sex score resulting in a new risk score (**Table 2**). Based on the calibration plots for NEED data (**Fig. 1**) and for the DMC (**Fig. 2**), the NEWS + age + sex was chosen as the best fit because calibration improved visually and according to the slope and intercept in the relevant risk range for all age categories while discrimination was not affected by adding sex. The NEWS + age + sex model was therefore proposed as the IEWS. Flexible calibration curves are shown in **Figures E3** and **E4** (http://links.lww.com/CCM/H313) for NEED data and DMC. Whereas the NEWS showed substantial underestimation of risk in older patients and overestimation of risk in younger patients, calibration for IEWS improved in both the development and validation cohort (Figs. 1 and 2).

**TABLE 2.** The International Early Warning Score

Points	5	3	2	1	0	1	2	3	5
Respiratory rate (breaths/min)					0–20		21–24	≥ 25	
Peripheral oxygen saturation (%)		≤ 91		92–95	≥ 96				
Supplemental oxygen				Yes	No				
Temperature (°C)	≤ 35.0	35.1–36.0			≥ 36.1				
Systolic blood pressure (mm Hg)			≤ 90	91–110	111–219		≥ 220		
Pulse (beats/min)				≤ 50	51–90	91–110	≥ 111		
Level of consciousness					Alert				Verbal, pain, or unresponsive
Sex				Male	Female				
Age, yr					0–40	41–50	51–60	61–65	76–80=5
								(66–75= 4 points)	(81–90 = 6 points)
									(91–100= 7 points)

The International Early Warning Score is a recalibrated model of the National Early Warning Score extended with age and sex.

Overall, AUROC improved substantially for IEWS with 0.89 (95% CI, 0.89–0.90) compared with NEWS 0.82 (95% CI, 0.82–0.83) in the NEED and in the DMC with AUROC for IEWS 0.87 (95% CI, 0.85–0.88) compared with NEWS 0.82 (95% CI, 0.75–0.89). For most age categories, discrimination improved substantially (**Table 3**). Internal validation showed good performance of IEWS (**Fig. E5**, http://links.lww.com/CCM/H313). Split sample analyses based on hospital location showed similar results (**Table E5**, http://links.lww.com/CCM/H313).

**TABLE 3.** Calibration and Discrimination for National Early Warning Score and International Early Warning Score in the Development and Validation Cohort

Age Groups	Calibration		Discrimination
	Intercept	Slope	Area Under the Receiving Operating Curve (95% CI)
Development cohort			
NEWS for in-hospital mortality in the NEED			
18–65 yr	–0.68	1.32	0.87 (0.85–0.88)
66–80 yr	0.38	0.97	0.80 (0.79–0.81)
> 80y	0.97	0.91	0.78 (0.77–0.80)
Overall	0.18	1.09	0.82 (0.82–0.83)
IEWS for in-hospital mortality in the NEED			
18–65 yr	0.15	1.47	0.92 (0.90–0.93)
66–80 yr	0.21	1.23	0.85 (0.84–0.86)
> 80 yr	0.16	1.18	0.83 (0.82–0.85)
Overall	0.18	1.24	0.89 (0.89–0.90)
Validation cohort			
NEWS for in-hospital mortality in the DMC			
18–65 yr	–1.05	1.09	0.82 (0.75–0.89)
66–80 yr	0.43	0.82	0.78 (0.74–0.82)
> 80 yr	0.94	0.84	0.78 (0.74–0.81)
Overall	0.20	0.98	0.82 (0.80–0.84)
IEWS for in-hospital mortality in the DMC			
18–65 yr	–0.52	1.05	0.86 (0.80–0.91)
66–80 yr	–0.04	0.88	0.80 (0.76–0.83)
> 80 yr	–0.25	0.83	0.77 (0.73–0.81)
Overall	–0.21	0.94	0.87 (0.85–0.88)

DMC = Danish Multicenter Cohort, IEWS = International Early Warning Score, NEED = Netherlands Emergency Department Evaluation Database, NEWS = National Early Warning Score.

The IEWS is a recalibrated model of the NEWS including the additional variables age and sex.

**Figure 3.**
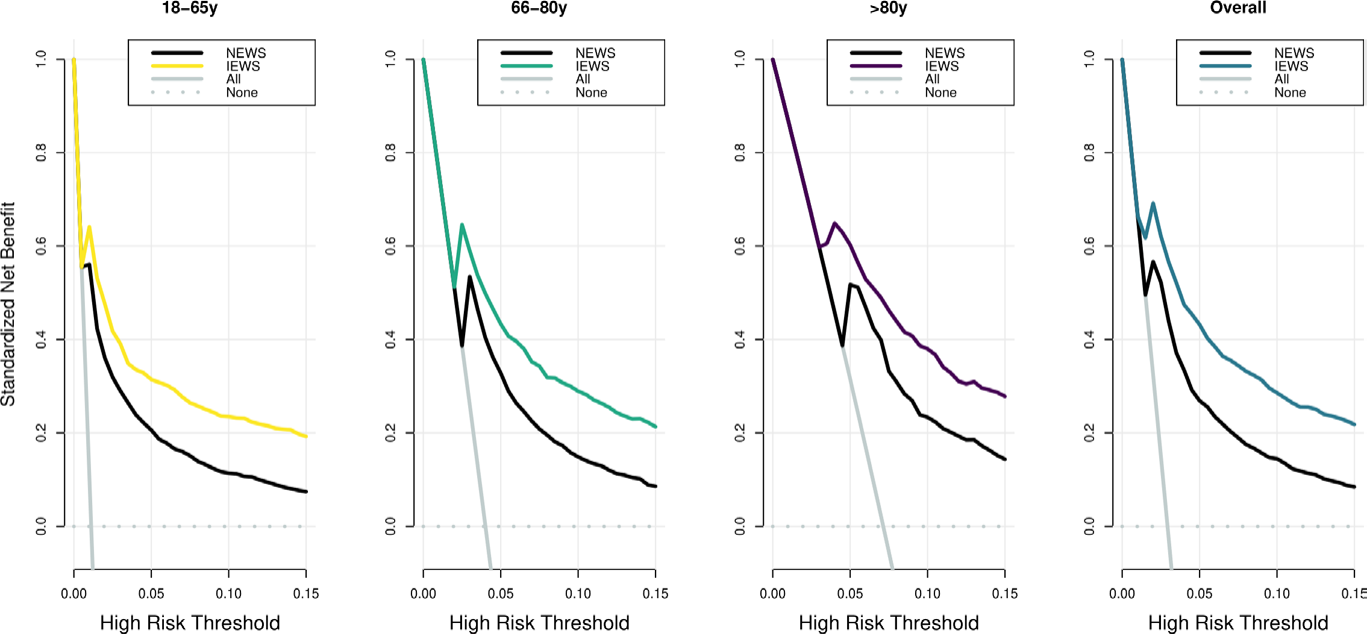
Decision curve analyses showed for each age category a standardized net benefit of 5–15% in the relevant risk range of 1–15%. As an example, in a population with approximately 24 in-hospital deaths per 1,000 patients, for a decision threshold of 5% in-hospital mortality risk, the International Early Warning Score (IEWS) would identify 42% additional true deaths (standardized net benefit at a threshold of 5% for IEWS), without increasing the number of false positive predictions compared with not using any model. Compared with using National Early Warning Score (NEWS), the IEWS would identify 15% additional true deaths without increasing the number of false positive predictions.

Decision curve analyses showed for each age category a standardized net benefit of 5–15% in the relevant risk range of 1–15% (**Fig. 3**). As an example, in a population with approximately 24 in-hospital deaths per 1,000 patients, for a decision threshold of 5% in-hospital mortality risk, the IEWS would identify 42% additional true deaths (standardized net benefit at a threshold of 5% for IEWS in Fig. 3), without increasing the number of false positive predictions compared with not using any model. Compared with using NEWS, the IEWS would identify 15% additional true deaths without increasing the number of false positive predictions. In the validation cohort, the net benefit improved similarly except in the younger age category in whom mortality was very low (**Fig. E6**, http://links.lww.com/CCM/H313).

To give a better insight in the benefit of using IEWS compared with NEWS, a reclassification table is presented in **Figure E7** (http://links.lww.com/CCM/H313).

## DISCUSSION

This large international multicenter cohort study shows that the IEWS, a recalibrated model based on NEWS including age and sex, performs significantly better compared with the widely adopted NEWS for the prediction of in-hospital mortality in ED patients of all age categories in a development and external validation cohort.

Most EWSs have methodological weaknesses that could have detrimental effects on patient care if used in clinical practice (19, 27). For example, NEWS, based on the VitalPAC early warning score, did not include age because AUROC only slightly improved after including age (15, 28). However, calibration, a key aspect of prediction model performance (16, 19), has not been assessed and age was used as a dichotomous variable below or above 65 years instead as continuous predictor. Furthermore, points for vital sign disturbances were allocated based on clinical consensus rather than on a statistical approach (1, 15).

Our results are in line with several studies which have demonstrated that including age to an EWS improved predictive performance substantially (9, 28–30). However, none of these studies followed the recommended steps for development and validation of prediction models neither have they shown a classification in low to high risk (31, 32). Our decision curve analysis and reclassification table demonstrate that for both younger and older patients the IEWS has considerable incremental value with more young patients correctly classified as low risk, and more importantly, more older patients correctly classified as high risk for in-hospital mortality. Previous studies have shown that predictive performance only improved for younger patients using an age-specific EWS on a composite outcome of mortality, cardiac arrest, and ICU admission compared with NEWS (33–35). However, the modeling approach was very different from ours. Points were assigned to vital signs based on their distribution rather than on regression coefficients as recommended for prediction modeling (19, 20, 31). This may have caused the age-specific model to underperform in older age. Our group has demonstrated previously that the addition of age to NEWS without recalibration of the physiologic variables already improved predictive performance for in-hospital mortality (10).

EWSs are designed for prognostication and can be used as early as in the ED and add to the clinical evaluation of a patient’s disease severity (36). While clinical evaluation may vary among physicians depending on years of experience (37), the IEWS provides a numerical mortality risk (a percentage) that hypothetically may help with clinical decision-making.

For an easily adopted and implemented EWS it is essential that the variables in the score are easily measured, readily available and strong predictors of the primary outcome (22, 31). The physiologic variables used in NEWS meet all these requirements (11, 12). In addition, age and sex exhibit the same qualities and are predictors for in-hospital mortality (10, 13). Other variables have been proposed to use in EWSs, such as biomarkers or frailty measures (38–42). For frailty, only four out of 60 frailty scores could be measured in less than 1 minute using vignettes (43). Although, in clinical practice, it may be difficult and not reliable to assess frailty, for example, if the level of consciousness is altered and no history is available. Other variables such as biomarkers are not readily available or easily repeated without high costs. For these reasons, we have only evaluated age and sex as additional variables to the seven predictors of NEWS which both met the criteria for reliable predictors and are always known or can at least be estimated precisely (19, 31). In patients who received prehospital treatments from paramedics or medical emergency services, the physiologic variables may already be improved at arrival to the ED and thus the risk may be underestimated by using the NEWS or IEWS, a phenomenon called lead-time bias in literature (44). Prehospital treatments have not been considered in the model. However, the IEWS still performs better overall than NEWS also in the ED.

The present study has several strengths. We adhered to the TRIPOD guidelines and followed steps recommended for the development of prediction models (16, 19, 20, 31). We used a large sample size in relation to the number of predictors for both development and validation and validated our findings externally in a different European country in a different time-period. The IEWS is clinically useful in the relevant risk range for each age category. Further validation is desired to assess generalizability of the proposed IEWS across multiple settings (45, 46). Other limitations need to be considered. First, a risk of selection bias may be present as we excluded patients in whom less than two vital signs were registered. However, these patients were at very low risk of mortality (e.g., wounds and fractures) or at very high risk (cardiac arrest) and therefore these patients would have been recognized as low or high risk also without an EWS. As recommended, we used multiple imputation to prevent information bias so we could include as many patients as possible (19). Notably, around 90% of AVPU values were missing in the development cohort. However, missingness was clearly related to outcomes and other measured variables (**Table E6**, http://links.lww.com/CCM/H313), IEWS worked very well in the external validation cohort with a very low missingness of the AVPU variable. Hence, the bias incurred by imputing AVPU is likely negligible, despite a high proportion of missingness (47). Second, in-hospital mortality was chosen as the primary outcome. A time horizon of a few days only is recommended for EWSs (19). Nevertheless, the time till patients died in-hospital in our data was short with a median of 4 days (IQR, 1–9 d), which allowed us to compare our results with previous studies and assess deterioration of patients (4, 5, 48). Third, the physiologic variables have been categorized based on the NEWS because physicians are used to work with these thresholds in clinical practice. However, it has been recommended to avoid categorizing predictors in the statistical analysis. For this reason, we repeated our analysis using restricted cubic splines for each physiologic variable and presented a nomogram (**Fig. E8**, http://links.lww.com/CCM/H313). Using this nomogram would have resulted in similar distribution of points as in the IEWS after rounding. Thus, categorization of variables did not lead to poor modeling. Nonetheless, using different points for each physiologic variable may lead to calculation errors as physicians are used to using the NEWS. This could be overcome by calculating the score electronically. Last, the NEWS2, a modification of the original NEWS, has not been evaluated in this study for several reasons. First, mortality prediction did not improve using NEWS2 compared with NEWS in a previous large study (49). Second, the two major updates introduced in NEWS2 were separate thresholds for saturation in patients with hypercapnic failure and the addition of confusion in consciousness scale. We did not record confusion, which makes it impossible to use the NEWS2 consciousness scale. Additionally, information about current or previous hypercapnic failure is often not available at arrival or requires arterial blood gas. We therefore bases the IEWS on the foundation laid out in NEWS rather than NEWS2. Comparing the IEWS with other widely adopted EWSs, such as the Modified Early Warning Score (MEWS), would have resulted in similar results, as the design of MEWS was neither based on a statistical approach, nor it includes age or sex (29).

In summary, this large international multicenter cohort study shows that the IEWS performs substantially better than the widely adopted NEWS for predicting mortality in ED patients of all age categories in a development and external validation cohort. Future studies should investigate further evidence for predictive validity and assess whether implementation of IEWS in the ED leads to lower adverse events compared with not using an EWS or using NEWS.

## Supplementary Material


