# Supplementary material for: Development and External Validation of the International Early Warning Score for Improved Age- and Sex-Adjusted In-Hospital Mortality Prediction in the Emergency Department
Source: Crit Care Med. 2023 Mar 23;51(7):881–91. doi: 10.1097/CCM.0000000000005842 (PMC10262984; doi:10.1097/CCM.0000000000005842)
Supplement: Supplementary file 1 [file ccm-51-881-s001.docx]

Authors: Bart Gerard Jan Candel, Søren Kabell Nissen, Christian H. Nickel, Wouter Raven, Wendy Thijssen, Menno I Gaakeer, Annmarie Touborg Lassen, Mikkel Brabrand, Ewout W Steyerberg, Evert de Jonge, Bas de Groot.

**Online-only supplemental**

Table E1 Patient characteristics of excluded patients in NEED P. 2

Table E2 Patient characteristics of excluded patients in DMC P. 3

Appendix 1 P. 4

Table E3 Patient characteristics P. 5

Table E4 Patient characteristics in the Danish Multicenter Cohort (DMC) used for external validation. P. 7

Figure E1 The association between age and in-hospital mortality P. 8

Figure E2 Nomogram for a recalibrated National Early Warning Score +age+sex P. 9

Figure E3 Flexible calibration plots in the NEED P. 11

Figure E4 Flexible calibration plots in the DMC P. 12

Figure E5 Internal validation P. 13

Table E5 Split sample analysis based on hospital location in the NEED P. 14

Figure E6 Decision Curve Analysis in the DMC P. 15

Figure E7 Reclassification figure and table for the development cohort. P. 16

Table E6 Multivariable logistic regression for missing glasgow coma scale P. 17

Figure E8 Nomogram for vital signs used as restricted cubic splines. P. 18

**Table E1** **Patient characteristics of excluded patients.**

Patient characteristics of both included (0-3 missing vitals) and excluded patients (4-5missing vitals) are presented in the table for comparison. Patients with four or five missing vital signs (Systolic blood pressure, temperature, heart rate, peripheral oxygen saturation or respiratory rate) were excluded from the analyses, because vital signs were considered missing not at random in these patients. Excluded patients are described in the table.

Excluded patients had lower in-hospital mortality, less ICU admissions and lower urgency of triage compared to included patients.

|  |  | **Included patients Excluded patients**  **Number of missing vital signs** | | | | | | |
| --- | --- | --- | --- | --- | --- | --- | --- | --- |
| **NEED cohort** | 0 (N=50378) | | 1 (N=29174) | 2 (N=10383) | 3 (N=5618) |  | *4 (N=5843)* | *5 (N=47393)* |
| **In-hospital mortality** | | | | | |  |  |  |
| died | 1406 (2.8%) | | 614 (2.1%) | 215 (2.1%) | 79 (1.4%) |  | *57 (1.0%)* | *478 (1.0%)* |
| Missing | 681 (1.4%) | | 437 (1.5%) | 210 (2.0%) | 159 (2.8%) |  | *148 (2.5%)* | *519 (1.1%)* |
| **ICU admission** | | | | | |  |  |  |
| ICU admission | 821 (1.6%) | | 549 (1.9%) | 143 (1.4%) | 48 (0.9%) |  | *31 (0.5%)* | *310 (0.7%)* |
| No ICU admission | 49195 (97.7%) | | 28267 (96.9%) | 9965 (96.0%) | 4770 (84.9%) |  | *5145 (88.1%)* | *39976 (84.4%)* |
| Missing | 362 (0.7%) | | 358 (1.2%) | 275 (2.6%) | 800 (14.2%) |  | *667 (11.4%)* | *7107 (15.0%)* |
| **Triage category*** | | |  |  |  |  |  |  |
| immediate | 3376 (6.7%) | | 1370 (4.7%) | 396 (3.8%) | 110 (2.0%) |  | *86 (1.5%)* | *632 (1.3%)* |
| very urgent | 17015 (33.8%) | | 7761 (26.6%) | 2218 (21.4%) | 665 (11.8%) |  | *516 (8.8%)* | *4913 (10.4%)* |
| urgent | 20768 (41.2%) | | 12855 (44.1%) | 4731 (45.6%) | 2927 (52.1%) |  | *2515 (43.0%)* | *16502 (34.8%)* |
| non-urgent | 8552 (17.0%) | | 6600 (22.6%) | 2825 (27.2%) | 1835 (32.7%) |  | *2595 (44.4%)* | *20845 (44.0%)* |
| Missing | 667 (1.3%) | | 588 (2.0%) | 213 (2.1%) | 81 (1.4%) |  | *131 (2.2%)* | *4501 (9.5%)* |

*Triage category according to the Manchester Triage System or Dutch Triage Standard.

**Table E2 Patient characteristics of excluded patients in DMC**

Patients were excluded if neither systolic blood pressure nor pulse were recorded as these observations were missing not at random, i.e., unrelated to any of the observed variables, including outcomes.

| **DMC Cohort** | **Included**  **N=14809** | | | | | | **Excluded**  **N=2039** | | | | **Pearson χ2** |
| --- | --- | --- | --- | --- | --- | --- | --- | --- | --- | --- | --- |
|  | **Number of missing vital signs (frequency)** | | | | | | | | | | |
|  | 0  (9132) | 1  (4075) | 2  (1357) | 3  (217) | 4  (21) | 5  (7) | 3  (4) | 4  (7) | 5  (201) | 6  (1827) |  |
| **Inhospital mortality, N (%)** | 215 (2.4) | 105 (2.6) | 36 (2.7) | 8 (3.7) | 1 (4.8) | 0 (0) | 1 (25.0) | 0 (0) | 7 (3.5) | 50 (2.7) |  |
| Subtotal | 365 (2.5) | | | | | | 59 (2.8) | | | | χ2=1.06, P=0.304 |
| **ICU admission, N (%)** | 220 (2.4) | 142 (3.5) | 46 (3.4) | 8 (3.7) | 1 (4.8) | 0 (0) | 1 (25.0) | 1 (14.3) | 7 (3.5) | 51 (2.8) |  |
| Subtotal | 417 (2.8) | | | | | | 60 (2.9) | | | | χ2=0.1, P=0.746 |

Missing vital signs include respiratory rate, heart rate, systolic blood pressure, peripheral oxygen saturation, temperature and avpu.

**Appendix 1**

**Sample size estimation**

The NEED contained 148,828 ED visits of patients ≥18 years. We estimated that in ~60% of the ED visits at least two or more vital signs were registered resulting in ~90,000 ED visits which could be used for the analyses with approximately 2300 events (in-hospital mortality). This number is more than sufficient for reliable analyses.(1) Numbers were also large in age-based subgroups. For external validation a minimum of in total 200 events is recommended.(1, 2) The DMC contains approximately 350 events which should be sufficient for external validation.(3)

**Multiple imputation procedure**

Because patients were included if at least two vital signs were registered, missing data in the NEED were substituted by multiple imputation to reduce information bias.(4) We used the chained equations procedure, after imputation was deemed feasible based on patterns of missingness.(5, 6) For a better multiple imputation procedure, we also used triage category (non-urgent, urgent, very urgent, immediate; according to the Manchester Triage System or Dutch Triage Standard), urea, leukocytes, and fluid administration (0, 0-500ml, >500ml) as a predictor in the imputation procedure. Outcome was imputed if missing. Imputation parameters for DMC have been described in detail previously.(3) We obtained 20 estimates of the missing vital signs for each patient with five iterations each. We checked for collinearity and convergence during the imputation procedure.

**Internal and external validation**

For internal validation, a bootstrapping was performed with 200 repetitions on the imputed data and the overall AUROC and calibration were presented.(7) Also, a non-randomly split sample analysis was performed based on hospital location in the NEED cohort.

The DMC was used for external validation. Predictive performance in terms of discrimination and calibration was assessed and based on the average estimate of risk. A net benefit curve was produced.

References:

1. Steyerberg EW. Clinical prediction models: Springer; 2019.

2. Collins GS, Ogundimu EO, Altman DG. Sample size considerations for the external validation of a multivariable prognostic model: a resampling study. Stat Med. 2016;35(2):214-26.

3. Nissen SK, Candel BG, Nickel CH, et al. The Impact of Age on Predictive Performance of National Early Warning Score at Arrival to Emergency Departments: Development and External Validation. Ann Emerg Med. 2021.

4. Harrell FE. Regression modeling strategies: with applications to linear models, logistic and ordinal regression, and survival analysis: Springer; 2015.

5. Azur MJ, Stuart EA, Frangakis C, et al. Multiple imputation by chained equations: what is it and how does it work? Int J Methods Psychiatr Res. 2011;20(1):40-9.

6. Vergouwe Y, Royston P, Moons KG, et al. Development and validation of a prediction model with missing predictor data: a practical approach. J Clin Epidemiol. 2010;63(2):205-14.

7. Steyerberg EW, Bleeker SE, Moll HA, et al. Internal and external validation of predictive models: a simulation study of bias and precision in small samples. J Clin Epidemiol. 2003;56(5):441-7.

**Table E3 Patient characteristics**

| **NEED cohort** | **18-65years (N=51573)** | **66-80years (N=29591)** | **>80years (N=14389)** | **All (N=95553)** |
| --- | --- | --- | --- | --- |
| **Age, years** |  |  |  |  |
| Mean (SD) | 45.7 (14.0) | 72.9 (4.21) | 85.8 (3.87) | 60.1 (19.4) |
| **Sex** |  |  |  |  |
| male | 25424 (49.3%) | 16363 (55.3%) | 6316 (43.9%) | 48103 (50.3%) |
| **Systolic Blood Pressure (mmHg)** | | |  |  |
| Mean (SD) | 128 (28.2) | 137 (33.2) | 142 (34.3) | 133 (31.3) |
| Missing | 4380 (8.5%) | 1525 (5.2%) | 559 (3.9%) | 6464 (6.8%) |
| **Heart rate (bpm)** |  |  |  |  |
| Mean (SD) | 87.0 (20.7) | 85.7 (21.6) | 83.4 (20.8) | 86.0 (21.0) |
| Missing | 7037 (13.6%) | 2647 (8.9%) | 1232 (8.6%) | 10916 (11.4%) |
| **Respiratory Rate (/min.)** |  |  |  |  |
| Median [IQR] | 16.0 [14.0-19.0] | 17.0 [15.0-21.0] | 18.0 [15.0-22.0] | 17.0 [14.0-20.0] |
| Missing | 16800 (32.6%) | 7103 (24.0%) | 3226 (22.4%) | 27129 (28.4%) |
| **Peripheral oxygen saturation (%)** | | |  |  |
| Mean (SD) | 97.8 (2.96) | 96.4 (3.81) | 96.0 (3.84) | 97.1 (3.47) |
| Missing | 2443 (4.7%) | 1397 (4.7%) | 702 (4.9%) | 4542 (4.8%) |
| **Level of consciousness** |  |  |  |  |
| Alert | 4311 (8.4%) | 3290 (11.1%) | 1840 (12.8%) | 9441 (9.9%) |
| Verbal | 178 (0.3%) | 118 (0.4%) | 114 (0.8%) | 410 (0.4%) |
| Pain | 109 (0.2%) | 44 (0.1%) | 36 (0.3%) | 189 (0.2%) |
| Unresponsive | 57 (0.1%) | 42 (0.1%) | 15 (0.1%) | 114 (0.1%) |
| Missing | 46918 (91.0%) | 26097 (88.2%) | 12384 (86.1%) | 85399 (89.4%) |
| **Temperature (degrees Celcius)** | | |  |  |
| Median [IQR] | 37.0 [36.5-37.4] | 36.8 [36.5-37.4] | 36.8 [36.4-37.3] | 36.9 [36.5-37.4] |
| Missing | 9820 (19.0%) | 5161 (17.4%) | 2762 (19.2%) | 17743 (18.6%) |
| **Supplemental Oxygen** |  |  |  |  |
| yes | 1421 (2.8%) | 2130 (7.2%) | 1449 (10.1%) | 5000 (5.2%) |
| Missing | 15931 (30.9%) | 6984 (23.6%) | 2672 (18.6%) | 25587 (26.8%) |
| **Fluid administration (ml)** |  |  |  |  |
| 0cc | 24458 (47.4%) | 16065 (54.3%) | 7807 (54.3%) | 48330 (50.6%) |
| 0-500cc | 4593 (8.9%) | 2850 (9.6%) | 1496 (10.4%) | 8939 (9.4%) |
| >500cc | 5248 (10.2%) | 3231 (10.9%) | 1377 (9.6%) | 9856 (10.3%) |
| Missing | 17274 (33.5%) | 7445 (25.2%) | 3709 (25.8%) | 28428 (29.8%) |
| **Triage category, N (%)** | * |  |  |  |
| non-urgent | 11076 (21.5%) | 5683 (19.2%) | 3053 (21.2%) | 19812 (20.7%) |
| urgent | 22703 (44.0%) | 12510 (42.3%) | 6068 (42.2%) | 41281 (43.2%) |
| very urgent | 14413 (27.9%) | 9071 (30.7%) | 4175 (29.0%) | 27659 (28.9%) |
| immediate | 2541 (4.9%) | 1871 (6.3%) | 840 (5.8%) | 5252 (5.5%) |
| Missing | 840 (1.6%) | 456 (1.5%) | 253 (1.8%) | 1549 (1.6%) |
| **Urea (mmol/L)** |  |  |  |  |
| Median [IQR] | 4.9 [3.8-6.2] | 6.7 [5.3-9.0] | 8.2 [6.2-11.4] | 5.8 [4.4-7.9] |
| Missing | 11273 (21.9%) | 4435 (15.0%) | 2375 (16.5%) | 18083 (18.9%) |
| **Leukocytes (*10^9)** |  |  |  |  |
| Median [Min, Max] | 9.2 [7.0-12.0] | 9.0 [6.9-12.1] | 9.2 [7.1-12.2] | 9.1 [7.0-12.1] |
| Missing | 10667 (20.7%) | 4001 (13.5%) | 1921 (13.4%) | 16589 (17.4%) |
| **In-hospital mortality, N(%)** | | | | |
| Died | 488 (0.9%) | 981 (3.3%) | 845 (5.9%) | 2314 (2.4%) |
| Missing | 608 (1.2%) | 554 (1.9%) | 325 (2.3%) | 1487 (1.6%) |
| **ICU admission, N (%)** |  |  |  |  |
| ICU admission | 828 (1.6%) | 582 (2.0%) | 151 (1.0%) | 1561 (1.6%) |
| Missing | 1385 (2.7%) | 319 (1.1%) | 91 (0.6%) | 1795 (1.9%) |

SD: Standard deviation, IQR: Interquartile Range, ICU: Intensive Care Unit

*Triage category according to the Manchester Triage System or Dutch Triage Standard.

**Table E4 Patient characteristics in the Danish Multicenter Cohort (DMC) used for external validation.**

| **DMC cohort** | **<65**  **(N=7990)** | **65-80**  **(N=4316)** | **>80**  **(N=2503)** | **All**  **(N=14809)** |
| --- | --- | --- | --- | --- |
| **Age** |  |  |  |  |
| Mean (SD) | 44.9 (13.9) | 73.0 (4.4) | 86.1 (4.1) | 60.0 (20.0) |
| **Gender** |  |  |  |  |
| male | 4,186 (52.4%) | 2,146 (49.7%) | 1,352 (54.0%) | 7,684 (51.9%) |
| **Systolic blood pressure (mmHg)** |  |  |  |  |
| Mean (SD) | 135 (21.6) | 140 (26.2) | 140 (27.4) | 137 (24.1) |
| Missing | 21 (0.3%) | 11 (0.3%) | 12 (0.5%) | 44 (0.3%) |
| **Heart Rate (bpm)** |  |  |  |  |
| Mean (SD) | 84.8 (19.6) | 84.6 (20.3) | 81.6 (19.6) | 84.2 (19.8) |
| Missing | 54 (0.7%) | 40 (0.9%) | 29 (1.2%) | 123 (0.8%) |
| **Respiratory Rate (/min)** |  |  |  |  |
| Median [IQR] | 16 [5] | 16 [5] | 18 [8] | 16 [6] |
| Missing | 2,340 (29.3%) | 1,073 (24.9%) | 624 (24.9%) | 4,037 (27.3%) |
| **Peripheral oxygen saturation (%)** |  |  |  |  |
| Mean (SD) | 97.1 (3.1) | 95.4 (4.2) | 94.9 (4.7) | 96.2 (3.8) |
| Missing | 343 (4.3%) | 159 (3.7%) | 135 (5.4%) | 637 (4.3%) |
| **Level of consciousness** |  |  |  |  |
| Alert | 6,891 (86.2%) | 3,712 (86.0%) | 2,039 (81.5%) | 12,642 (85.4%) |
| Vocal | 101 (1.3%) | 84 (1.9%) | 84 (3.4%) | 269 (1.8%) |
| Pain | 36 (0.5%) | 23 (0.5%) | 22 (0.9%) | 81 (0.5%) |
| Unresponsive | 23 (0.3%) | 10 (0.2%) | 8 (0.3%) | 41 (0.3%) |
| Missing | 939 (11.8%) | 487 (11.3%) | 350 (14.0%) | 1,776 (12.0%) |
| **Temperature (Degrees Celsius)** |  |  |  |  |
| Median [IQR] | 37.0 [0.8] | 36.9 [0.9] | 36.9 [0.9] | 37.0 [0.8] |
| Missing | 526 (6.6%) | 234 (5.4%) | 182 (7.3%) | 942 (6.4%) |
| **Supplementary oxygen** |  |  |  |  |
| Yes | 353 (4.4%) | 427 (9.9%) | 309 (12.3%) | 1,089 (7.4%) |
| **In-hospital mortality, N (%)** |  |  |  |  |
| Died | 46 (0.6%) | 156 (3.6%) | 163 (6.5%) | 365 (2.5%) |
| **ICU admission, N (%)** |  |  |  |  |
| ICU admission | 197 (2.5%) | 144 (3.3%) | 76 (3.0%) | 417 (2.8%) |

**Figure E1 The association between age and in-hospital mortality**


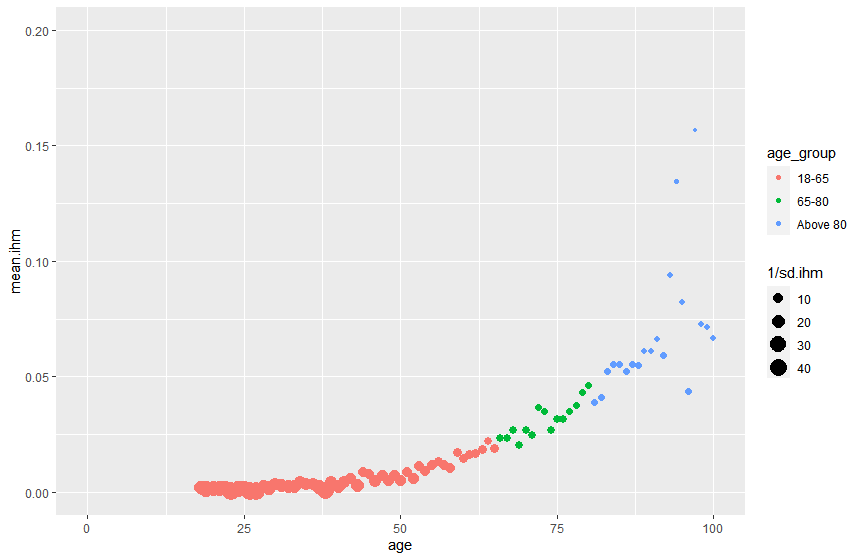


Ihm: in-hospital mortality

Included patients from the NEED (N=95,553). Age was used as a linear predictor in the prediction model above 40years old. The size of the dots indicates the precision of the estimate for observed in-hospital mortality and is based on the inverse of the standard deviation. The larger, the higher the precision.

**Figure E2** **Nomogram for a recalibrated National Early Warning Score +age+sex**


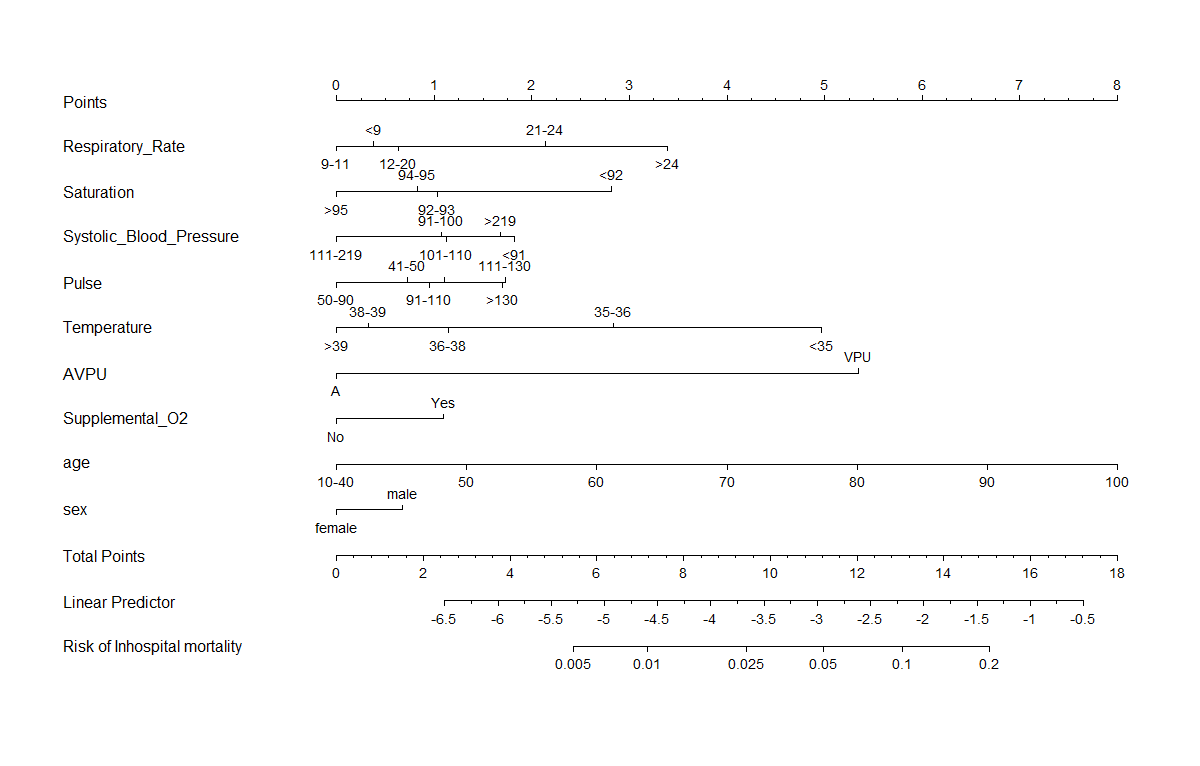


All predictors were used as categorized variables as used in the original National Early Warning Score (NEWS). A nomogram was developed based on the regression coefficients of all predictors (see below). A nomogram is a graphical presentation of model fit, allowing points to be awarded on a scale that is proportional to the log-odds. Points were rounded to nearest integer.

A nomogram fitted on the primary outcome (in-hospital mortality). As can be seen in the nomogram, age was the strongest predictor for mortality followed by AVPU. Male sex was associated with increased mortality. The nomogram for a recalibrated NEWS score with age and without sex was comparable to the recalibrated NEWS with age and with sex.

**Regression coefficients and odds ratio’s of multivariable logistic regression**

| Variables | Regression coefficients | Odds ratio’s (95% CI) | Variables | Regression coefficients | Odds Ratio’s  (95% CI) |
| --- | --- | --- | --- | --- | --- |
| Intercept | -1,45 | - | Pulse (<41bpm) | -0,22 | 1.5 (0.9-2.4) |
| RR (<9/min.) | 0,4 | 0.84 (0.35-2.0) | Pulse (41-50bpm) | 0,31 | 1.4 (1.0-1.9) |
| RR (9-11/min.) | -0,19 | 0.82 (0.54-1.6) | Pulse (51-90bpm) | 0 | Ref. |
| RR (12-20/min.) | 0 | Ref. | Pulse (91-110bpm) | -0,84 | 1.5 (1.3-1.6) |
| RR (21-24/min.) | 1,01 | 1.87 (1.6-2.2) | Pulse (110-130bpm) | -0,23 | 2.0 (1.7-2.3) |
| RR (>24/min.) | 1,91 | 3.1 (2.7-3.6) | Pulse(>130bpm) | -0,83 | 2.0 (1.6-2.5) |
| SPO2(>95%) | 0 | Ref. | Temp(<35dgr) | 0,14 | 4.7 (3.4-6.7) |
| SpO2(94-95%) | 0,35 | 1.4 (1.2-1.6) | Temp(35-36dgr) | 0,71 | 2.0 (1.7-2.4) |
| SPO2(92-93%) | -0,29 | 1.5 (1.3-1.8) | Temp(36-38dgr) | 0 | Ref. |
| SPO2(<92%) | 0,083 | 3.1 (2.7-3.6) | Temp(38-39dgr) | -2,44 | 0.72 (0.60-0.87) |
| SBP (>219mmHg) | -0,98 | 2.1 (1.5-3.1) | Temp(>39dgr) | -3,3 | 0.62 (0.48-0.80) |
| SBP(111-219mmHg) | 0 | Ref. | Alert | 0 | Ref. |
| SBP (101-110mmHg) | 0,43 | 1.5 (1.3-1.8) | VPU | 0,72 | 8.8 (7.4-10.5) |
| SBP(91-100mmHg) | -0,43 | 1.5 (1.3-1.8) | O2 (No) | 0 | Ref. |
| SBP(<91mmHg) | -0,58 | 2.1 (1.8-2.4) | O2 (yes) | 0,23 | 1.6 (1.4-1.8) |
| Age (counting from 63) | -0,95 | 4.5 (4.1-5.0) /28y | Sex (female) | -0,29 | 0.74 (0.68-0.82) |

**Function**

function(Rr2 = 0,Sat = 0,Sbp2 = 0,Pulse2 = 0,Temp2 = 0,Avpu = 0,O2 = 0,age = 63,sex = "male") {Rr2 <- ordered(Rr2);Sat <- ordered(Sat);Sbp2 <- ordered(Sbp2);Pulse2 <- ordered(Pulse2);Temp2 <- ordered(Temp2);age <- Function(age2)(age);

-1.4534925-0.19432847*(RR== 9-11) +1.0141945*(RR==21-24) +0.40291742*(RR<=9) +1.9103567*(RR>=24)+0.34889429*(SPO2=94-95)-0.28768878*(Sat==92-93) +0.082739612*(Sat<92) +0.43450926*(SBP=101-110)-0.43269485*(Sbp=91-100)-0.57997328*(Sbp<91)-0.97877706*(Sbp>219)+0.30637857*Pulse(41-50)-0.21608917*(Pulse<41)-0.23371859*(Pulse= 110-130)-0.8381017*(Pulse=91-110)-0.83097791*(Pulse>130)+0.7070425*Temp(35-36)+0.14159026*(Temp<35)-2.4456138*(Temp2=38-39)-3.2986931*(Temp>39)+0.72456848*vpu+0.23021481*O2(yes)-0.94793297*age-0.29486964*(sex=="female") }

**Figure E3** **Flexible calibration plots in the NEED**

**
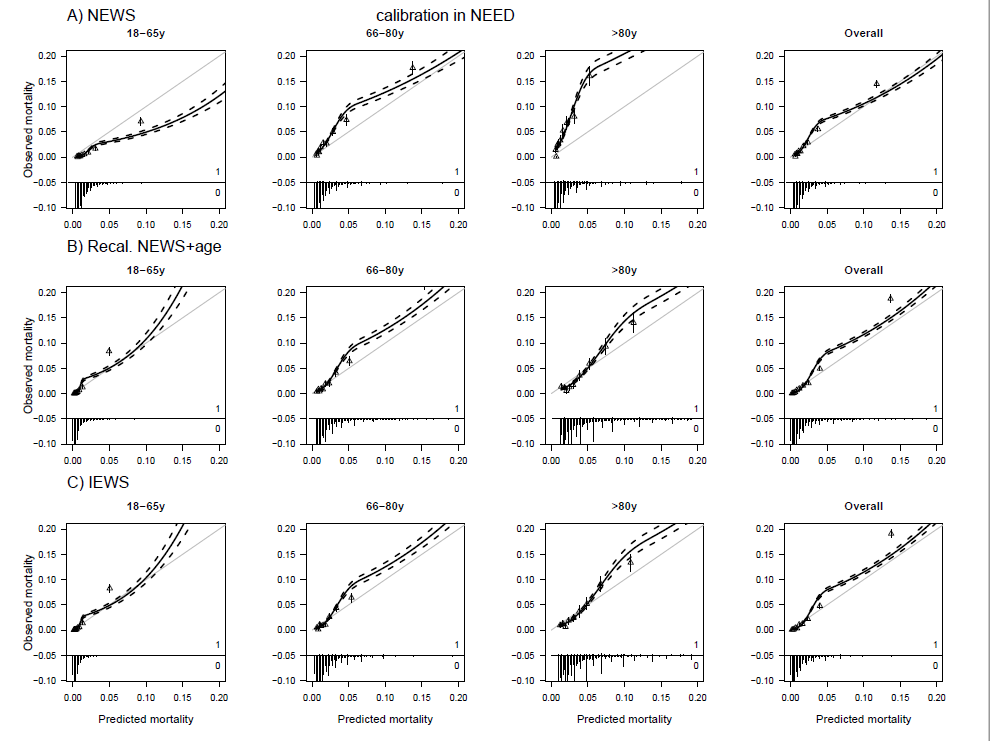
**

**Figure E4** **Flexible calibration plots in the DMC**


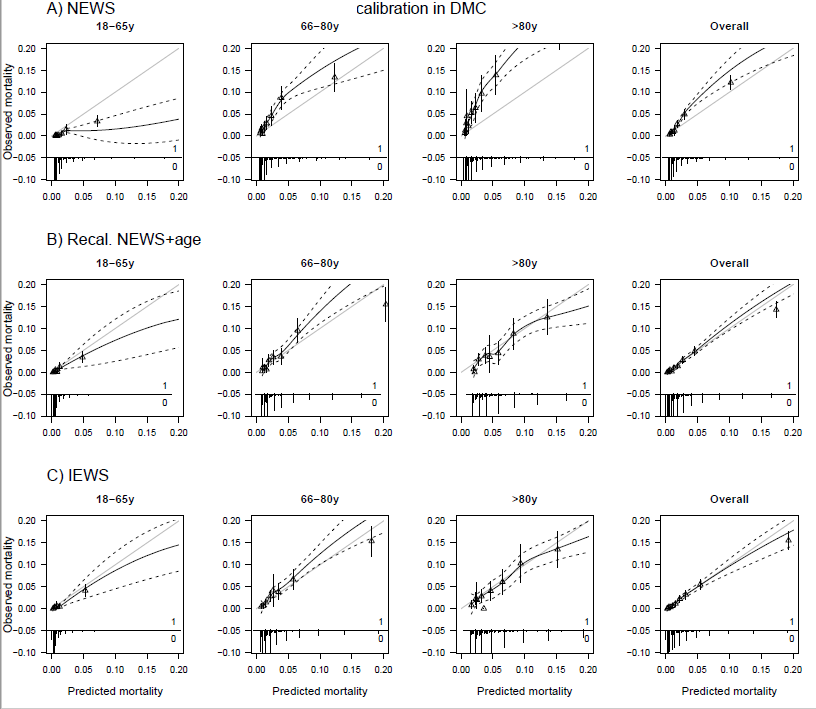


**Figure E5 Internal validation**

Internal validation using bootstrap with 200 random sample repetitions on the NEED.


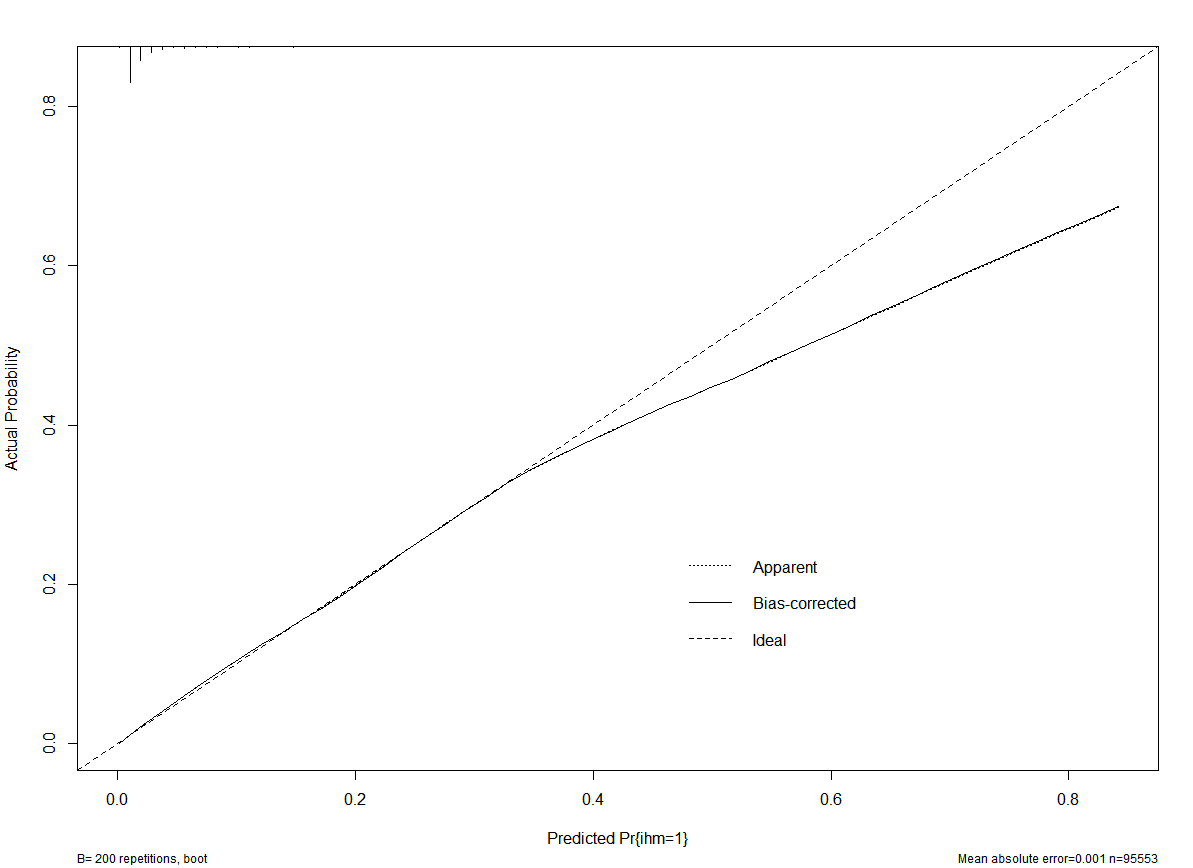
AUROC overall = 0.87

Calibration plot. Notes: Apparent refers to apparent performance for calibration; bias-corrected refers to optimism-corrected in internal validation; bootstrap=200.

**Table E5** **Split sample analysis based on hospital location in the NEED.**

|  | **Discrimination** | |
| --- | --- | --- |
| **Age groups** | **AUROC** | **95% CI** |
| **NEWS for in-hospital mortality** |  |  |
| Hospital 1 (tertiary care center) | 0.80 | 0.78-0.82 |
| Hospital 2 | 0.86 | 0.85-0.87 |
| Hospital 3 | 0.82 | 0.81-0.84 |
| **IEWS for in-hospital mortality** |  |  |
| Hospital 1 (tertiary care center) | 0.87 | 0.86-0.89 |
| Hospital 2 | 0.90 | 0.90-0.91 |
| Hospital 3 | 0.87 | 0.86-0.89 |

NEWS: National Early Warning Score, IEWS: International Early Warning score, AUROC: Area under the Receiving Operating Curve, 95% CI: 95percent Confidence Intervals, NEED: Netherlands Emergency Department Evaluation Database.

The International Early Warning Score is a recalibrated model of the NEWS including age and sex as extra variables.

The figure below is a calibration plot for NEWS compared to IEWS stratified by hospital location.

Hospital 1 (yellow), Hospital 2 (green), hospital 3 (purple)


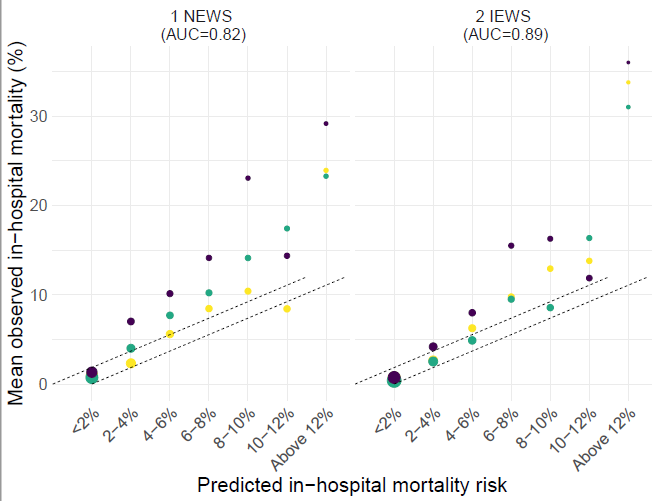


**Figure E6 Decision Curve Analysis in the DMC**


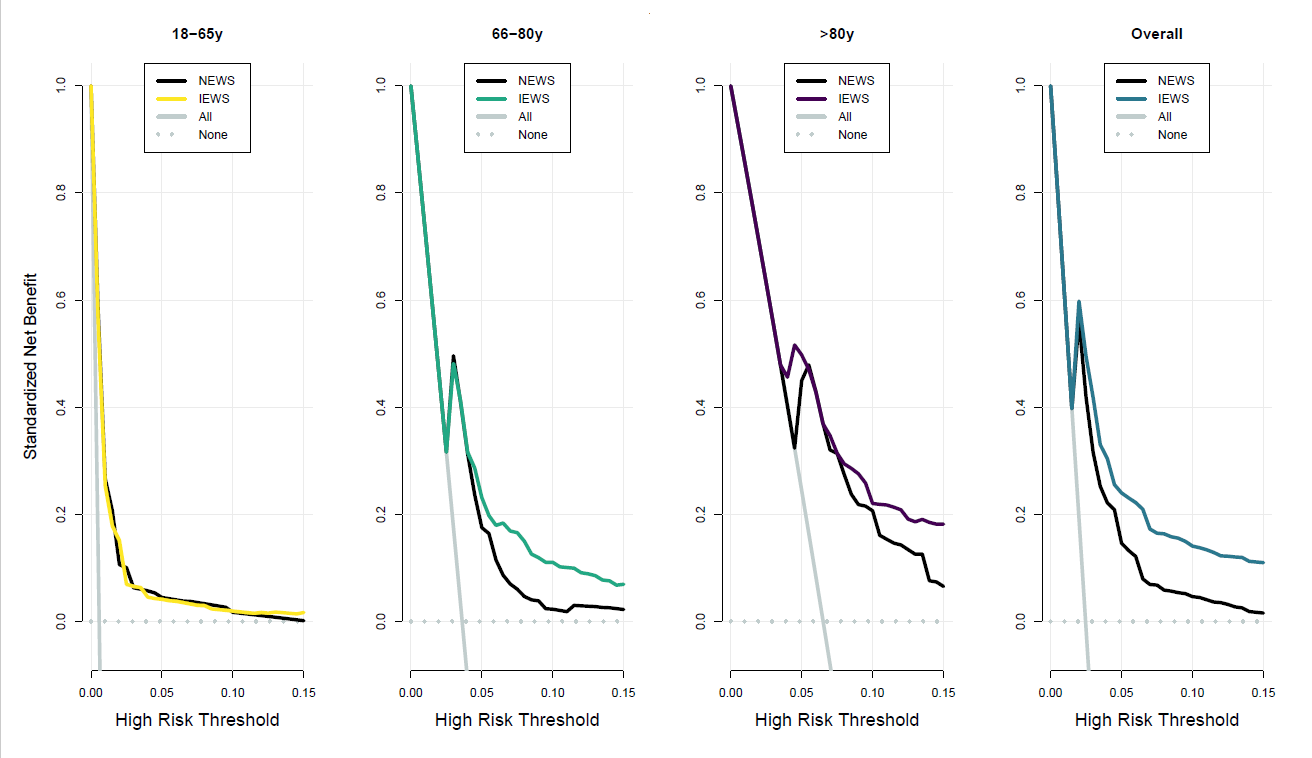


**Figure E7 Reclassification figure and table for the development cohort.**

The reclassification figure and table are presented as an example to show the benefit of using IEWS compared to NEWS in terms of classification. We have arbitrary divided patients into three risk groups: Low risk, medium risk and high risk of inpatient mortality. For this example, the baseline risk was calculated for each age category by the average mortality risk for patients with a NEWS<4. Patients were considered as lows risk if mortality was lower than 2*baseline risk, medium risk if mortality was between 2*baseline risk and 3*baseline risk, and high risk if mortality was more than 3*baseline risk. These thresholds and risk categories were only used as an example to visualize the differences between IEWS and NEWS.

18-65y Low risk <0.68%, medium risk 0.68-1.02% and high risk >1.02%

66-80y Low risk <2.94%, medium risk 2.94-4.41% and high risk >4.41%


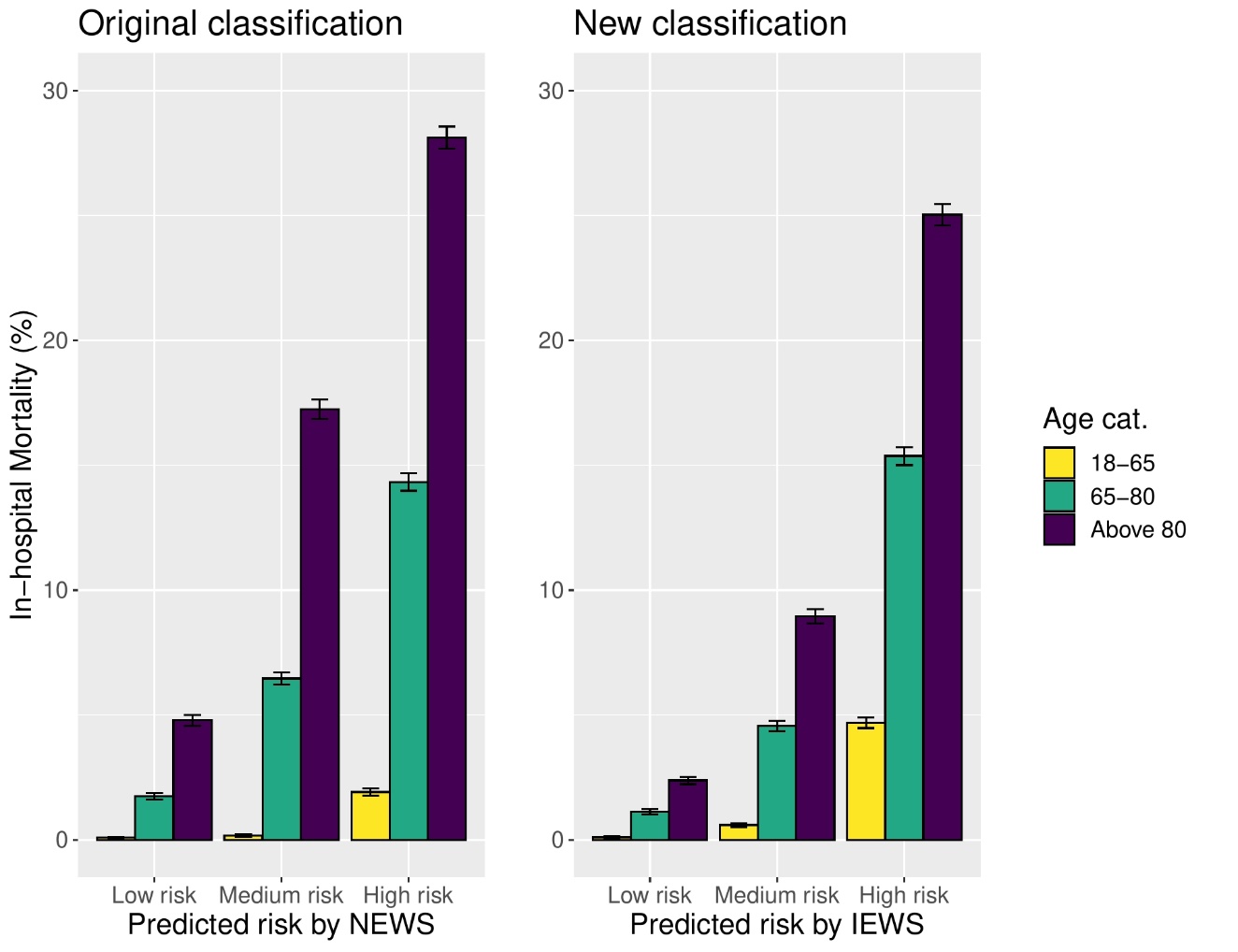

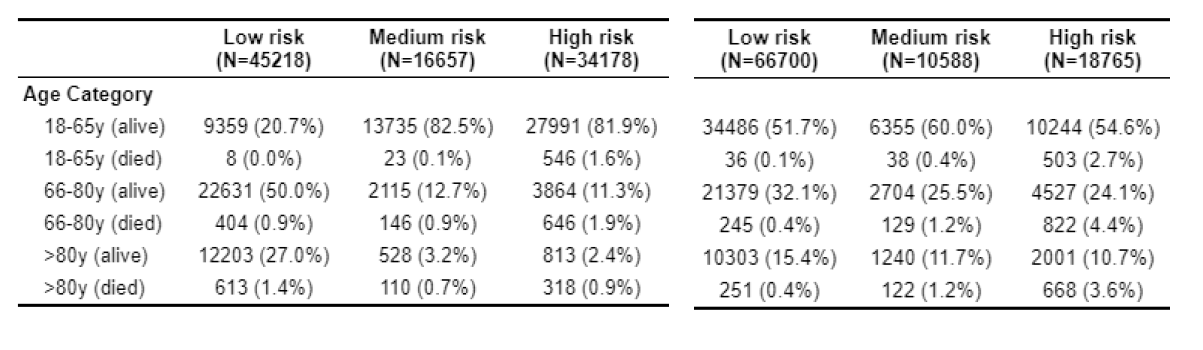
 >80y Low risk <5.96%, medium risk 5.96-8.94% and high risk >8.94%

As can be seen in the figure and table, using IEWS classifies more patients correctly as low risk for inpatient mortality compared to NEWS, and classifies more patients correctly as high risk compared to using NEWS. Overall, more younger patients were considered as low risk while more older patients were considered as medium or high risk. As a result, older patients who died in the hospital would have been recognized more precisely by using IEWS.

**Table E6 Multivariable logistic regression for missing glasgow coma scale**

A multivariable logistic regression model demonstrated that missing glasgow coma scale is associated with physiological variables and outcome (Chi-square p<0.01), and thus GCS is not missing completely at random or missing not at ramdom and is suitable for multiple imputation. Similar Chi-square p-values were found for the other physiological variables.

Logistic Regression Model

lrm(formula = is.na(gcs) ~ ihm + sbp + saturation + sex + triage +

age, data = NEED2)

Ratio Test

Obs         82306

FALSE       9290

TRUE       73016    Pr(> chi2) <0.0001

Coef    S.E.    Wald Z Pr(>|Z|)

Intercept        2.7829 0.3175    8.77 <0.0001

ihm                -0.2334 0.0630  -3.70 0.0002

sbp                -0.0095 0.0004 -26.49 <0.0001

saturation         0.0012 0.0032   0.37 0.7112

sex=female      0.0375 0.0225    1.67 0.0954

triage=very urgent  0.4236 0.0394  10.76 <0.0001

triage=urgent        0.9677 0.0397  24.37 <0.0001

triage=non-urgent    1.6921 0.0495  34.17 <0.0001

age                -0.0058 0.0007  -8.78 <0.0001

Frequencies of Missing Values Due to Each Variable

is.na(gcs)        ihm        sbp saturation        sex     triage        age

0       2154      59317      57074          2       6181          0

**Figure E8**

Nomogram for vital signs used as restricted cubic splines. Extremes for each vital sign were excluded.

**
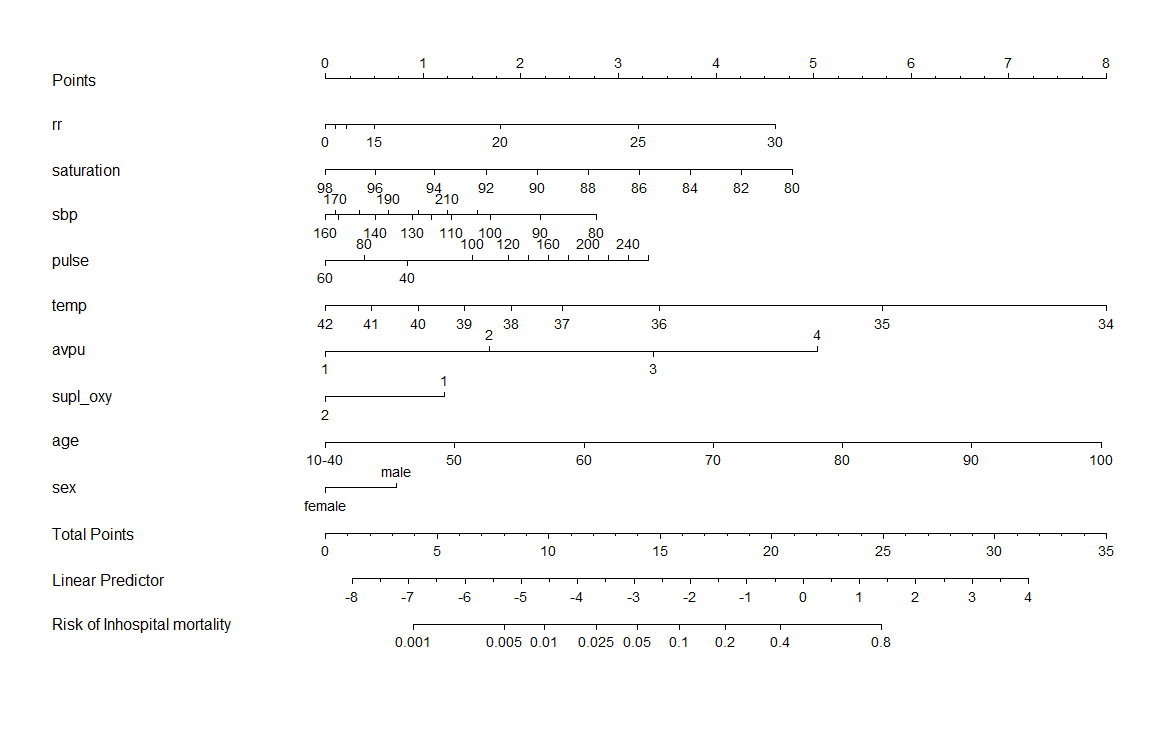
**
